# Synthesis of Mg-Al Hydrotalcite Clay with High Adsorption Capacity

**DOI:** 10.3390/ma14237231

**Published:** 2021-11-26

**Authors:** Zhaoyi Li, Jie Zhang, Chengtun Qu, Ying Tang, Michal Slaný

**Affiliations:** 1 State Key Laboratory of Petroleum Pollution Control, Xi’an Shiyou University, Xi’an 710065, China; 210104@xsyu.edu.cn (Z.L.); zhangjie@xsyu.edu.cn (J.Z.); xianquct@xsyu.edu.cn (C.Q.); 2 Shaanxi Key Laboratory of Carbon Dioxide Storage and Enhanced Oil Recovery, Xi’an 710060, China; 3 Shaanxi Province Key Laboratory of Environmental Pollution Control and Reservoir Protection Technology of Oilfields, Xi’an Shiyou University, Xi’an 710065, China; 4 Institute of Inorganic Chemistry, Slovak Academy of Sciences, Dúbravská cesta 9, 845 36 Bratislava, Slovakia; 5 Institute of Construction and Architecture, Slovak Academy of Sciences, Dúbravská cesta 9, 845 03 Bratislava, Slovakia

**Keywords:** metal oxides, filter paper, adsorption, sulfonated lignite, hydrotalcite, clay

## Abstract

A novel Mg-Al metal oxide has been successfully synthesized by the calcination of hierarchical porous Mg-Al hydrotalcite clay obtained by using filter paper as a template under hydrothermal conditions. Various characterizations of the obtained nanoscale oxide particles verified the uniform dispersion of Mg-Al metal oxides on the filter paper fiber, which had a size of 2–20 nm and a highest specific surface area (SSA) of 178.84 m^2^/g. Structural characterization revealed that the as-prepared Mg-Al metal oxides preserved the tubular morphology of the filter paper fibers. Further experiments showed that the as-synthesized Mg-Al metal oxides, present at concentrations of 0.3 g/L, could efficiently remove sulfonated lignite from oilfield wastewater (initial concentration of 200 mg/L) in a neutral environment (pH = 7) at a temperature of 298 K. An investigation of the reaction kinetics found that the adsorption process of sulfonated lignite (SL) on biomorphic Mg-Al metal oxides fits a Langmuir adsorption model and pseudo-second-order rate equation. Thermodynamic calculations propose that the adsorption of sulfonated lignite was spontaneous, endothermic, and a thermodynamically feasible process.

## 1. Introduction

Petroleum is the lifeblood of many industries; however, its extraction and processing—especially the development and construction of oil fields, due to drilling operations, pipeline laying, and other projects—will inevitably have an impact on the natural ecosystem. Sulfonated lignite is a good freshwater viscosity reducer and fluid loss reducer that can withstand high temperatures of 200–240 °C. SL is widely used in the application of drilling fluid, resulting in effects on the chemical oxygen demand (COD) and biochemical oxygen demand (BOD) of drilling wastewater [1,2], and millions of tons of high-concentration toxic organic drilling wastewater are produced during oilfield exploration in China every year. This often becomes a difficult problem for oilfield-produced water discharge, and it is very relevant to develop technologies to avoid further drilling fluid pollution. So far, a variety of methods, such as chemical precipitation, electrolysis, ion exchange, membrane separation, and adsorption, have been examined to remove pollutants from oilfield wastewater. Between these, adsorption technology has received the greatest attention due to the number of advantages, such as being relatively low-cost, highly efficient, simple, and eco-friendly. Various classes of adsorbents are utilized to remove pollutants from oilfield wastewater, including activated carbon, layered double hydroxides, metal oxides, and biosorbents. In recent years, metal oxide nanoparticles have become one of the most widely studied adsorption materials due to their large specific surface area with abundant active sites [3]. However, their larger specific area than traditional adsorbents, instability, single-pore structure, and their strong tendency for agglomeration limits their application range. Therefore, hierarchically porous materials with large surface area, low density, variable chemical compositions, large accessible space, and interconnected hierarchical porosity at different length scales have attracted considerable attention as important functional materials [4].

Layered double hydroxides (LDHs) are one of the attractive materials used to improve the adsorption activities of metal oxide materials [5]. Moreover, the calcined product of LDHs has a larger specific surface area and abundant pore structure. However, high calcination temperatures cause a collapse of the pore structure, and the particles agglomerate easily, which is not conducive to the improvement of the degree of activation of the metal oxides [6,7]. Although there are extensive studies on metal oxides, the preparation of a hierarchically porous LDO is still complicated and extremely challenging. Over billions of years, organisms have evolved and optimized, exhibiting natural hierarchical anatomies and morphologies with multi-scale dimensions and components [8]. In recent years, the preparation of nanomaterials by the plant template method has attracted widespread attention, as a material of choice can be deposited onto the templates to form a nanomaterial with the shape of the template. To date, various natural materials have been employed as templates to synthesize various biomorphic oxide materials, including wood rattan [9], cotton fibers [10], bamboo [11], eggshells [12,13], cellulose [14,15], and peanut shells [16,17], among others. In recent years, especially in the application of adsorption, studies on the preparation of metal oxides by biological template methods have also been of great interest. For example, Yu et al. synthesized ZnAl-LDH/ZnCo_2_O_4_ through a hydrothermal process with a pollen template, and the resulting material showed extraordinary adsorption ability in Congo red solution [18]. Using flax, gauze, and sisal as templates, Liu et al. successfully synthesized a ZnAl-CLDH/FeWO_4_ material which exhibited a high adsorption activity for the removal of dyes from water [19].

In this work, Mg-Al metal oxide composites were synthesized via a filter paper template under hydrothermal conditions to obtain hierarchically porous materials. The adsorption performance of the new adsorbents was investigated by looking at the removal efficiency of SL in oilfield wastewater.

## 2. Experimental

### 2.1. Materials

In this work, magnesium nitrate hexahydrate (Mg(NO_3_)_2_·6H_2_O) was bought from West Asia Chemical Technology Co. Ltd. Shandong, China. Urea (CO(NH_2_)_2_) and aluminum nitrate nonahydrate (Al(NO_3_)_3_·9H_2_O) were obtained from Damao Chemical Reagent Co., Ltd. Tianjin, China. Filter paper was obtained from the local market. Sulfonated lignite was purchased from Tarim, China. Ethanol was purchased from Fuyu Fine Chemical Co., Ltd. Tianjin, China. In addition, deionized water was used for all synthesis processes and for washing the precipitate.

### 2.2. Preparation of Mg-Al Metal Oxides Materials

**Figure 1:**
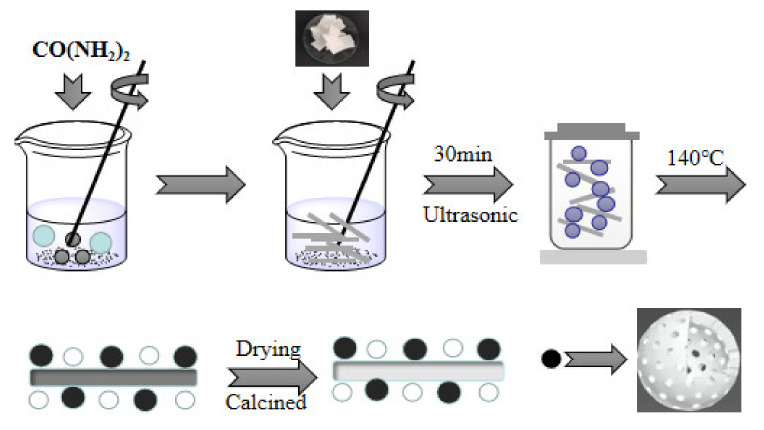
Preparation flow chart of Mg-Al metal oxide.

In our synthesis procedure (Figure 1), Mg-Al metal oxides were synthesized by a facile hydrothermal route. First, the filter paper was rinsed with deionized water and ethyl alcohol three times and then dried at 80 °C for 12 h for later use. Next, 4.10 g of Mg(NO_3_)_2_·6H_2_O, 3.01 g of Al(NO_3_)_3_·9H_2_O, and 4.36 g of urea were dispersed in 80 mL of deionized water at room temperature with vigorous stirring to form a mixed solution. After that, 1 g of the filter paper was added to the mixed solution under ultrasound for 30 min. The mixed solution was heated at 140 °C and transferred into a 500 mL autoclave pressure vessel for varying lengths of time. The as-prepared products were taken out of the vessel after cooling to room temperature, washed with distilled water 3 times, and dried in the drying furnace at 80 °C for 12 h. Eventually, the sample was calcined under 500 °C, 600 °C, and 700 °C in an air atmosphere for 3 h (ramping rate was 10/min). Besides that, a blank sample without a template using the same experimental process was prepared.

### 2.3. Characterization of Materials

Using a scanning electron microscope with a 20.0 kV accelerating voltage (SEM, JSM-6390A, JEOL, Tokyo, Japan) the morphology of the prepared material was elucidated. X-ray diffraction analysis (XRD), (JDX-3530, JEOL, Tokyo, Japan) using an X-ray copper tube was used for an investigation of the structures of the prepared material. Ka radiation release at accelerations to 40 mA and 40 kV were arranged at 10–90° 2θ degree with a scan speed of 2/min. Specific surface area (SSA) and pore structure measurements were carried out by liquid N_2_ (−196 °C) using a BET analyzer, Micromeritics ASAP 2010 (Norcross, GA, USA), at a temperature of 77 K. SSA calculation was based on the Brunauer–Emmett–Teller (BET) method. The Barrett–Joyner–Halenda (BJH) method was used for calculating the pore size distribution. FTIR spectra were collected using a Nicolet 5700 Fourier transform infrared (Thermo Electron Co., West Palm Beach, FL, USA) and 1 mg of the sample was homogenized with 200 mg of KBr to form powder pellets. A total of 64 scans with a resolution of 4 cm^−1^ were used for FTIR measurements.

### 2.4. Adsorption Experiment

This work used sulfonated lignite (SL) as a pollutant to study the adsorption capacity of the obtained materials. A certain amount of adsorbent was added to the 50 mL SL solution with an initial concentration of 200 mg·L^−1^. The mixture was stirred for 12 h to ensure the adsorption equilibrium at room temperature (303 K). Then, the solutions were filtered and the residual concentrations of SL were measured by a visible UV-vis spectrophotometer Avantes (Apeldoorn, The Netherlands) with a maximum wavelength of 300 nm and also ultraviolet radiation. The effects of different parameters—such as the amount of adsorbent and pH value—on the adsorption process in the solution were studied. With the addition of 0.1 mol·L^−1^ HCl or 0.1 mol·L^−1^ NaOH, the pH value of the solution was altered. To improve the accuracy of the adsorption findings to obtain average values, the experiments were repeated at least three times. Based on the formula given below, the amount of SL adsorbed was calculated:

(1) $$q_{t}=\frac{\left(C_{0}-C_{t}\right)V}{M}$$

(2) $$q_{e}=\frac{\left(C_{0}-C_{e}\right)V}{M}$$

where *C_e_* (mg·L^−1^) and *C*_0_ are the equilibrium and initial SL concentration, *M* is the mass of the adsorbent (g), and *V* is the volume of the solution (mL).

## 3. Results and Discussion

### 3.1. Structural Characterization

#### 3.1.1. SEM Analysis

**Figure 2:**
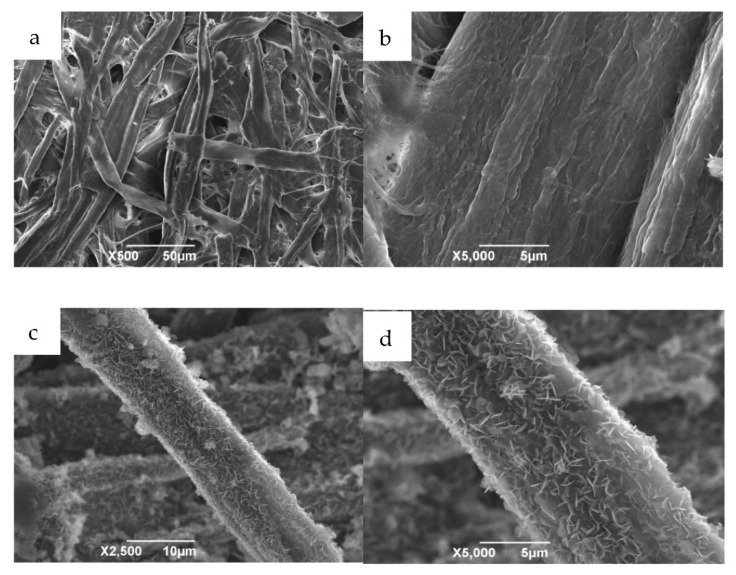

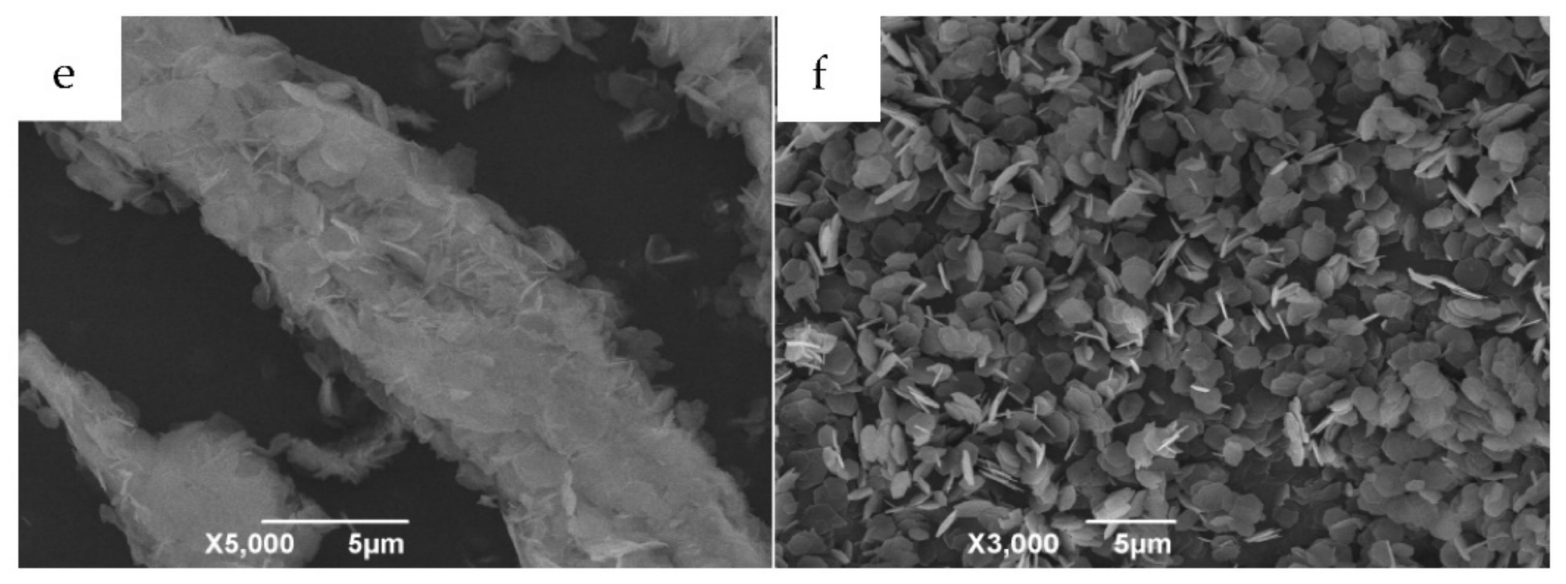
SEM images of filter paper (**a**,**b**); filter paper-templated Mg-Al LDH (**c**,**d**); filter paper-templated MgAl metal oxides (600 °C, 3 h) (**e**); Mg-Al metal oxides crystallized without a template (**f**).

Figure 2 shows the SEM micrographs of the as-prepared samples. Figure 2a,b are SEM images of the filter paper under different magnifications. It can be concluded that the microstructure of the filter paper is a three-dimensional network structure composed of many micron-sized nanotubes. Figure 2c,d show the morphology of Mg-Al LDHs derived from the filter paper template. In the hydrothermal processes, Mg^2+^ and Al^3+^ cations were dispersed on the filter paper surfaces; subsequently, the increase in temperature caused the decomposition of urea to produce OH^−^ and CO_3_^2−^, which led to the deposition of magnesium nitrate and aluminum nitrate, forming Mg-Al LDH on the filter paper templates.

Furthermore, it was found that the layer double hydroxide (LDH) nanoplatelets vertically aligned on the surface of the filter paper fibers in an orderly fashion, indicating that the mechanism of the crystal growth process is governed by self-assembly and in situ crystallization [20]. Moreover, the filter paper fiber shape is well-maintained, even upon increasing the calcination temperature to 600 °C, as shown in Figure 2e. These results demonstrate that the evolutionarily optimized microstructure of filter paper was inherited by the template-assisted synthesized Mg-Al metal oxides after the complete removal of the biotemplate. Figure 2f presents an image of Mg-Al metal oxides derived from Mg-Al LDH without the template. It is clearly visible from the figure that the Mg-Al metal oxides exhibited the flat, hexagonal, sheet-like morphology typical of such oxides.

#### 3.1.2. Specific Surface Area and Porosity Analysis

**Figure 3:**
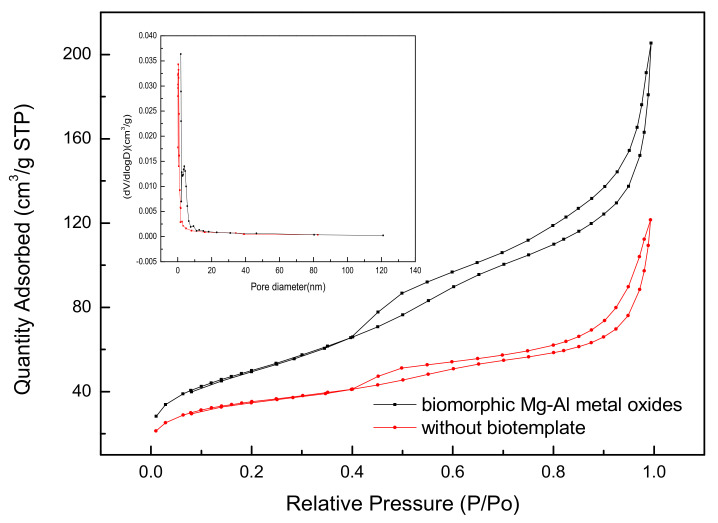
Nitrogen adsorption-desorption isotherms and corresponding BJH pore size distribution curves of the samples.

Calculating the specific surface area (SSA) using a BET analysis based on nitrogen adsorption–desorption isotherms is the most common way to identify the porosity of various materials [21]. In Figure 3, the N_2_ adsorption–desorption isotherms and pore size distribution curves of different samples can be seen. It was observed that the N_2_ adsorption–desorption isotherm of the Mg-Al metal oxides synthesized with the template exhibits a type IV isotherm with an H3-type hysteresis loop (P/P0 > 0.4), indicating that slit-like pores are formed by the accumulation of flake particles [22]. A sharp increase is seen at high pressure (P/P0 = 0.9–1.0). This higher adsorption at high relative pressure indicates that macropores are also present [23]. On the curve of the biomorphic Mg-Al metal oxides, it can be seen that the distribution range of the filter paper-templated particles is 1.78–120 nm. As illustrated in Table 1, the introduction of paper fiber templates causes a distinct increase in BET surface area, from 117.04 to 178.84 m^2^/g, and in pore volume from 0.19 to 0.32 cm^3^/g, which provides access to more adsorption sites by facilitating the diffusion of sulfonated lignite.

#### 3.1.3. XRD Analysis

**Figure 4:**
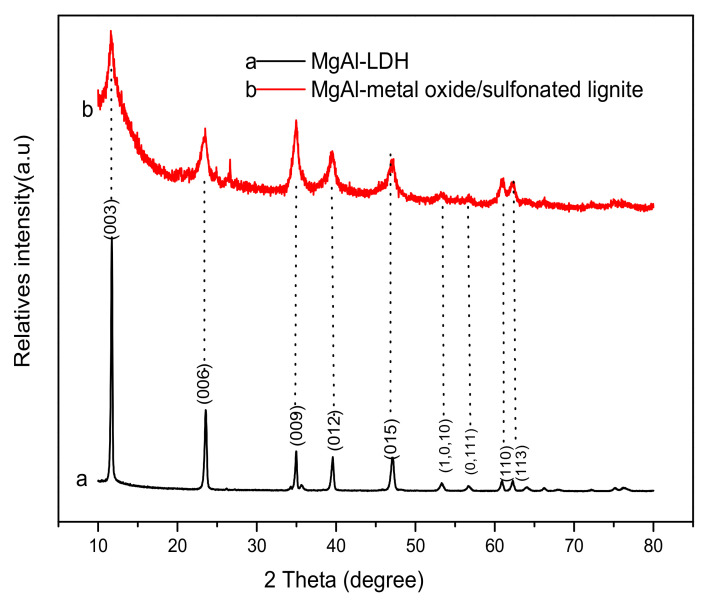
XRD patterns of (**a**) Mg-Al LDH and (**b**) Mg-Al metal oxides after the adsorption of sulfonated lignite.

**Figure 5:**
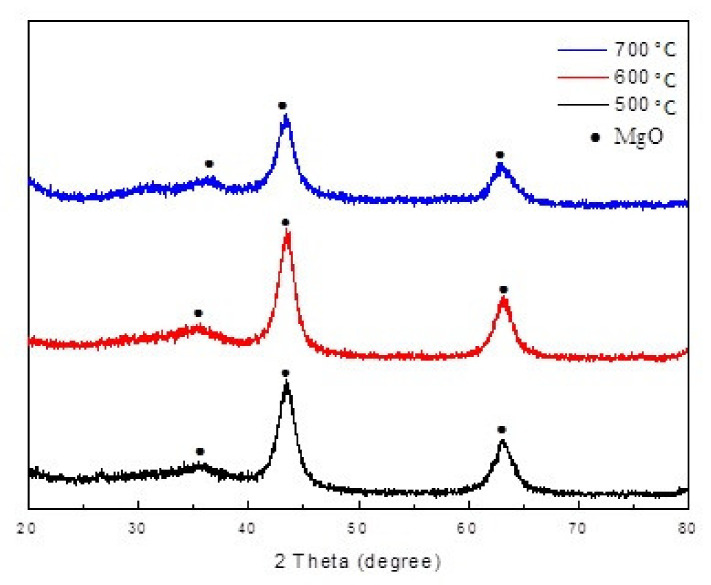
XRD pattern of filter paper-templated Mg-Al metal oxides.

Figure 4a and Figure 5 show the XRD pattern of Mg-Al metal oxides and Mg-Al LDH. The main diffraction peaks of MgAl-LDH (JPCDS, no.350964) can be assigned to the (003), (006), (012), (015), (018), (110), (113), and (116) diffraction planes. These findings propose that the synthesized LDH crystallized with well-ordered, layered structures. After calcination at 500–700 °C, diffraction peaks can be observed at 2θ values of about 36.9°, 42.9°, and 62.3°, which are respectively assigned to the (111), (200), and (220) reflections of MgO (JCPDS, no.450946) [22]. The characteristic diffraction peaks of MgAl-LDH disappeared, indicating that the layered structure of MgAl-LDH was converted and destroyed into aluminum and magnesium mixed oxides. A shortage of peaks belonging to aluminum oxide means that the Al^3+^ ions are well-dispersed in the MgO. The XRD pattern of Mg-Al metal oxides after the adsorption of sulfonated lignite is also shown in Figure 4b. The characteristic diffraction peaks of hydrotalcite are clearly visible. This indicates that the Mg-Al metal oxide has undergone the reconstruction process of hydrotalcite, which is also known as the “memory effect” of hydrotalcite.

#### 3.1.4. FTIR Analysis

**Figure 6:**
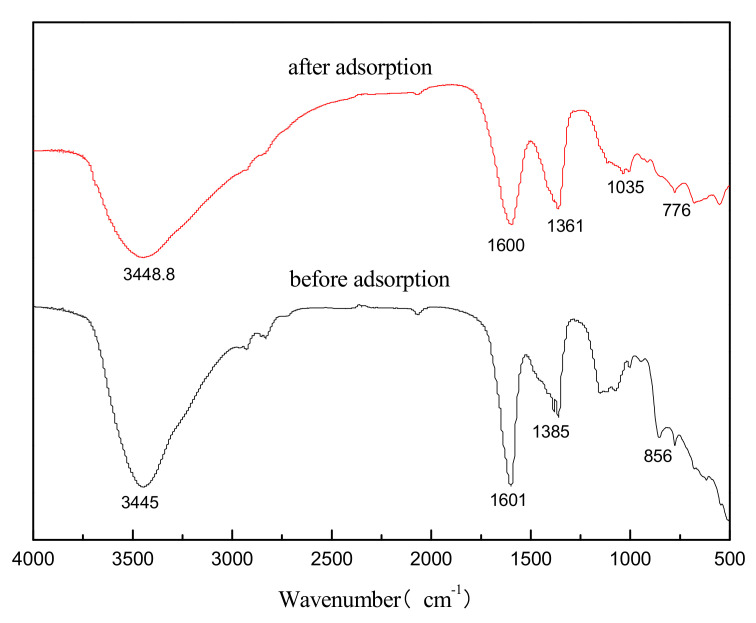
FTIR spectra of Mg-Al metal oxides before and after adsorption.

The FTIR spectra of Mg-Al metal oxides before and after the adsorption of sulfonated lignite are presented in Figure 6. A broad and strong complex band at 3445 cm^−1^ found for all samples is related to the stretching vibration of –OH groups, indicating from hydrogen-bonded, interlayered H_2_O molecules, or metal –OH groups. The absorption band at 1600 cm^−^^1^ is assigned to the –OH flexural groups of the interlayer crystal water, and the stretching vibration caused by the small peak carbonate is visible at 1361 cm^−^^1^. However, from the FTIR spectra of the Mg-Al metal oxides after adsorption, it can be seen that the crystal water and hydroxyl groups at 3445 cm^−^^1^ and 1600 cm^−^^1^ are significantly weakened, and an O=S=O stretching vibration band appeared at 1035 cm^−1^. This finding indicates that the Mg-Al metal oxides successfully adsorbed the sulfonated lignite molecules in the solution. A series of absorption bands recorded in the 770–860 cm^−1^ region were ascribed to M-O (M = Mg, Al) vibrations.

### 3.2. Adsorption Performance

#### 3.2.1. Comparison of Adsorbents

**Figure 7:**
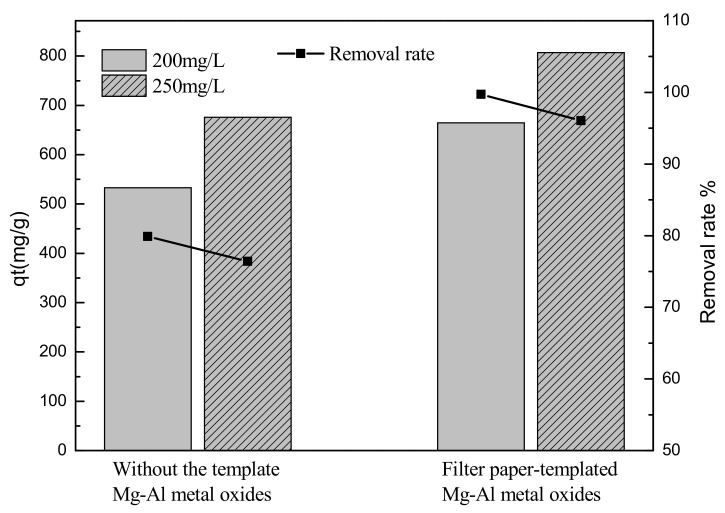
Comparison of the degree of adsorption of sulfonated lignite by filter paper-templated Mg-Al metal oxides and by untemplated Mg-Al metal oxides (initial sulfonated lignite concentration: 200 mg/L and 250 mg/L; adsorbent dosage: 0.015 g; initial pH: 7; temperature set to 25 °C; stirring time: 12 h).

Sulfonated lignite was chosen as the model pollutant to evaluate the adsorption capability of Mg-Al metal oxides. Figure 7 shows the adsorption capacities of the Mg-Al metal oxides derived from Mg-Al LDH, prepared with and without a filter paper template. The results are that a sulfonated lignite adsorption capacity of 664.83 mg/g and 807.06 mg/g was achieved for initial sulfonated lignite concentrations of 200 and 250 mg/L, respectively, by 0.3 g/L of Mg-Al oxides derived from hierarchical hydrotalcite at an initial pH of 7 and a temperature of 25 °C. Moreover, the adsorption capacities of Mg-Al metal oxides prepared without the use of a filter paper template were also investigated and found to have a lower sulfonated lignite adsorption capacity (525.43 mg/g and 665.34 mg/g, respectively). This difference can be attributed to the vertical matrix growth of MgAl-LDH nanosheets, which avoids the multilayer accumulation of nanosheets, gives the material a rich pore structure and a larger specific surface area, and provides more available active adsorption sites.

#### 3.2.2. Effect of Precursor Concentration, Reaction Time, Calcination Temperatures, Adsorbent Dosage, and pH

**Figure 8:**
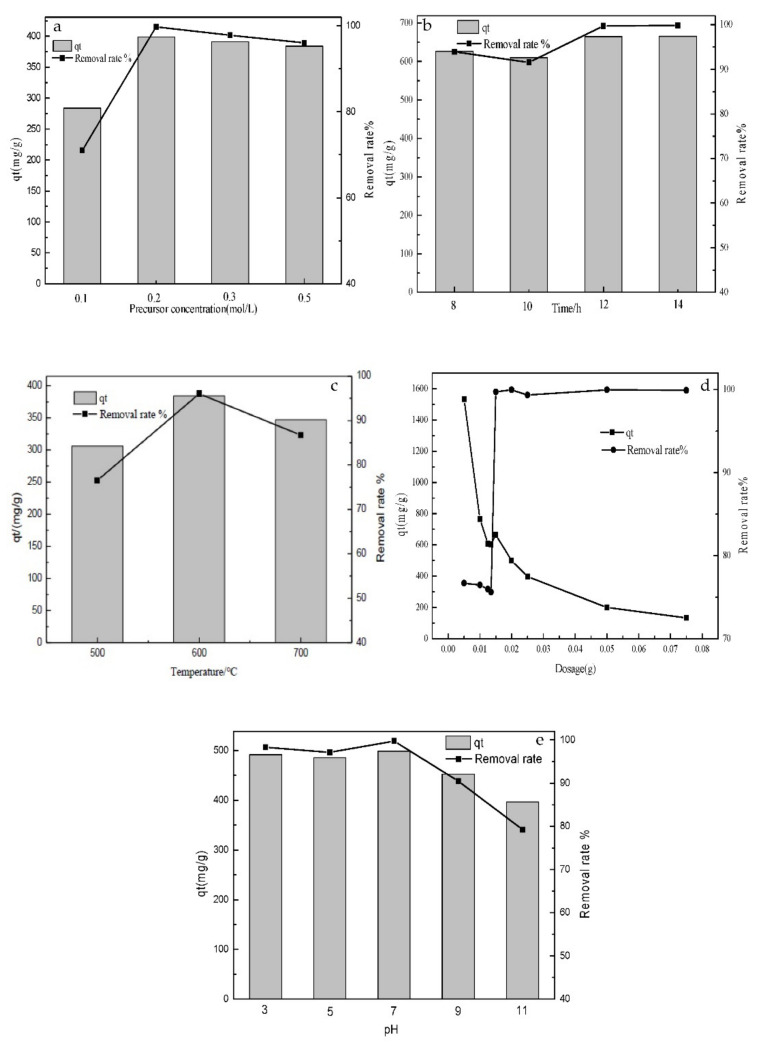
Effects of precursor concentration (mol/L) (**a**), reaction time (h) (**b**), calcination temperatures (°C) (**c**), adsorbent dose (g) (**d**), and pH (**e**) on the adsorption of sulfonated lignite by filter paper-templated Mg-Al metal oxides.

The effects of precursor concentration on the adsorption of sulfonated lignite onto filter paper-templated Mg-Al metal oxides are shown in Figure 8a. The experimental results indicate that the filter paper-templated Mg-Al metal oxides, prepared from a 0.2 mol/L precursor, exhibit the highest adsorption capacity, wherein the removal rate of SL reached 97.73%. By continuing to increase the concentration of the precursor solution, the removal rate of sulfonated lignite will decrease, due to the increase in the solution viscosity as the result of incomplete mixing with the template. This causes blocked channels in the high-temperature calcination process and decreases the surface area available for adsorption, which then leads to a decrease in removal efficiency.

The effect of reaction time on the adsorption of sulfonated lignite by filter paper-templated Mg-Al metal oxides is shown in Figure 8b. The removal percentage of sulfonated lignite increased from 93.94 to 99.73% when the reaction time was prolonged from 8 to 12 h. However, upon further increasing the reaction time, the adsorption capacity of sulfonated lignite remained unchanged, indicating that the adsorption had reached equilibrium.

The adsorption capacities of filter paper-templated Mg-Al metal oxides derived at different calcination temperatures (500, 600, and 700 °C) were compared under the same adsorption conditions and are shown in Figure 8c. It can be seen that when the roasting temperature is increased from 500 °C to 600 °C, the removal rate of sulfonated lignite is increased from 76.53% to 96.06%, while a decrease is seen upon further increasing the calcination temperature to 700 °C, due to the high calcination temperature causing a collapse of the pore structure, which is not conducive to improving the degree of activation of the adsorbent.

The dose of adsorbent is an important parameter that affects the efficiency of the removal process. In this study, the effect of the adsorbent dosage on sulfonated lignite adsorption efficiency was studied by varying the adsorbent dosage from 0.1 to 1.5 g/L at an initial sulfonated lignite concentration of 200 mg/L; the results are shown in Figure 8d. The removal rate of sulfonated lignite increases from 76.69 to 99.37% when the adsorbent dosage increases from 0.1 to 0.3 g/L, which could be attributed to the large number of active sites on the surface of the adsorbent at the initial stage of adsorption, and the sulfonated lignite molecules quickly occupy these active sites [23]. However, beyond the latter dosage, decreased performance was found, and the removal efficiency remained constant even up to an adsorbent dosage of 1.5 g/L, thus indicating that a higher adsorbent dose results in a lower uptake capacity. This is presumably due to the increase in the amount of adsorbent leading to the aggregation of solid particles, the overlapping of active sites, and the reduced adsorption capacity of the adsorbent.

Solution pH plays an important role in the adsorption process due to the extent of ionization or speciation of the adsorbate and its influence on the surface charge of the adsorbent [24]. To evaluate the effect of pH on sulfonated lignite removal efficiency, experiments were conducted at pH values of 3, 5, 7, 9, and 11 using filter paper-templated Mg-Al metal oxides, and the results are summarized in Figure 8e. The maximum adsorption was 498.63 mg/g, with a removal efficiency of 99.73% at a pH value of 7 in the presence of an initial sulfonated lignite concentration of 200 mg/L. The adsorbed amount and the removal rate of sulfonated lignite remained at a high level (485 mg/g and 97% or more) at a lower pH. We attribute this to the protonation of Mg-Al metal oxides at neutral or acidic pH, and thus the formation of positively charged M-OH_2_^+^ [25], which in turn causes electrostatic attraction between the positively charged metal oxide surface and the negatively charged adsorbent molecule [26]. In contrast, in the alkaline range (pH = 9, 11), sulfonated lignite adsorption decreases with higher pH. In addition to the lower degree of protonation on the surface, this may also be due to the competition of OH^−^ with sulfonated lignite ions [27]. In addition, the increase in the number of –OH groups reduces the number of positive charges on the surface of the adsorbent, thereby reducing its adsorption capacity for sulfonated lignite.

#### 3.2.3. Kinetics of Adsorption

To determine and interpret the mechanisms and kinetics of the sulfonated lignite adsorption process by filter paper-templated Mg-Al metal oxides, the experimental data obtained were fitted to pseudo-first-order [28,29] and pseudo-second-order kinetic models [30,31] and intra-particle diffusion models [32] according to the linear equations of these three kinetic models, as follows:

(3) $$\log\left(q_{e}-q_{t}\right)=\log q_{e}-\frac{k_{1}t}{2.303}$$

(4) $$\frac{t}{q_{t}}=\frac{1}{k_{2}q^{2}e}+\frac{t}{q_{e}}$$

(5) $$q_{t}=k_{di}\sqrt{t}+c_{i}$$

where *k*_1_ (min^−1^) and *k*_2_ (g/mg·min) are the rate constants of the pseudo-first-order and pseudo-second-order models, q_t_ is the amount of adsorption over time (t), and *k_di_* and *c_i_* are the intra-particle diffusion model rate parameter and the intercept of stage *i*, respectively.

**Figure 9:**
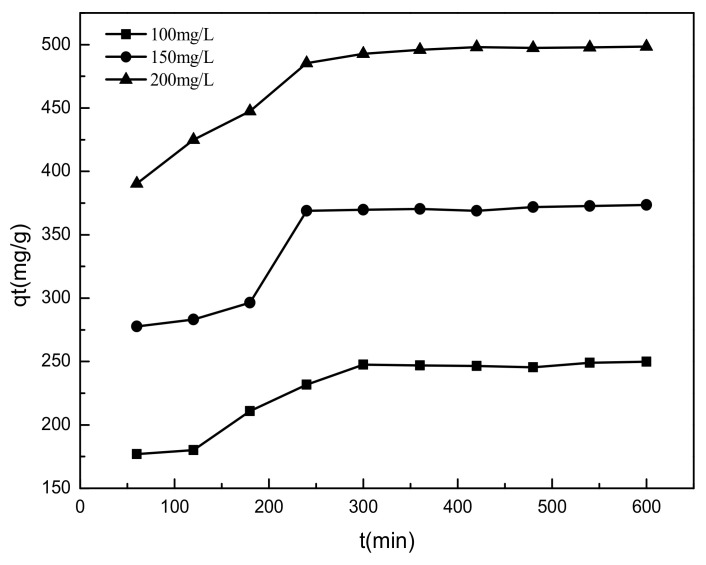
Effect of adsorption time on adsorption efficiency.

SL adsorption onto filter paper-templated Mg-Al metal oxides is plotted against contact time in solution in Figure 9, for initial sulfonated lignite concentrations of 100, 150, and 200 mg/L. For all initial concentrations, the adsorbed amount of sulfonated lignite increased rapidly at the beginning, due to plentiful vacant sites on the Mg-Al metal oxide surface, making the concentration gradient the driving force for adsorption. For a 200 mg/L initial concentration, the adsorption capacity of the sulfonated lignite was 484.14 mg/g at 240 min, constituting 97.08% of equilibrium adsorption capacity. After that, further adsorption was minimal, as the system was approaching equilibrium. Similar behavior was seen for the other initial concentrations.

**Figure 10:**
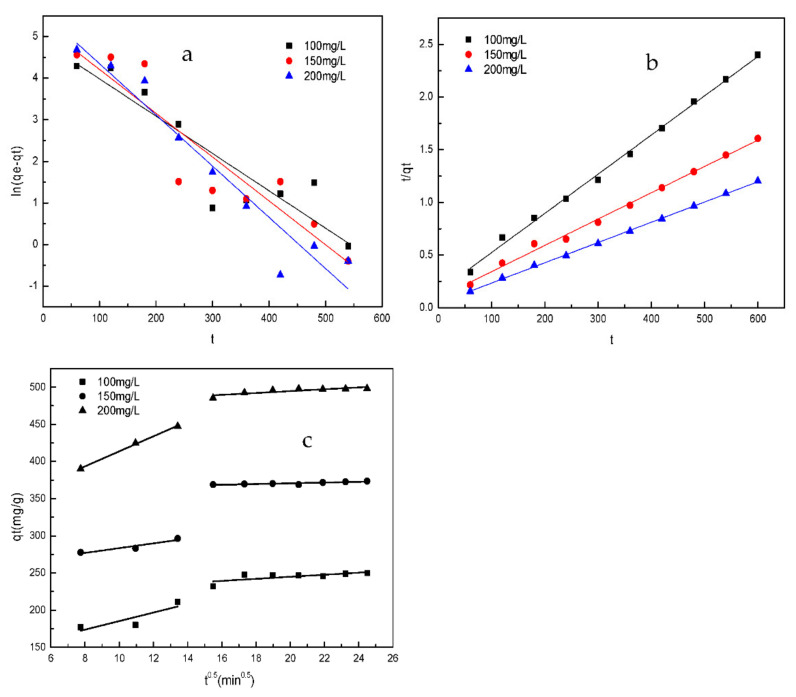
The fit of the pseudo-first-order (**a**), pseudo-second-order (**b**), and intra-particle diffusion (**c**) models for the adsorption of sulfonated lignite by filter paper-templated Mg-Al metal oxides.

Figure 10 illustrates the pseudo-first-order, pseudo-second-order, and intra-particle diffusion models of sulfonated lignite adsorption by filter paper-templated Mg-Al metal oxides. From the intercept and slope of the straight line obtained, the kinetic parameters for the removal of SL were determined, as listed in Table 2 and Table 3. Looking at the parameters, the pseudo-first-order model had a relatively lower correlation coefficient, and a large difference in the q_e_ value between the calculation (q_e_, cal) and the experiment (q_e_, exp) was noticed, confirming that the adsorption process did not follow the pseudo-first-order model. In contrast, the almost identical values of calculated and experimental q_e_, and the high values of R^2^ (>0.99), make the pseudo-second-order model a much better fit, which suggests that chemical adsorption was the main rate-limiting step in the adsorption of sulfonated lignite by the prepared Mg-Al metal oxides [33].

For the evaluation of the prediction and the mechanism of the rate-limiting steps, the intraparticle diffusion model was used. In Figure 10c, the plot exhibits a multi-straight-line nature, indicating the different stages involved in the adsorption process. The plots of q_t_ versus t^1/2^ give a straight line and none of the lines pass through the origin, which reveals that intra-particle diffusion is not the rate-controlling step for the whole adsorption process. Table 3 shows that the k_d1_ values are larger than the k_d2_ values, which suggests that the surface diffusion step was important for the adsorption of sulfonated lignite by the biomorphic Mg-Al metal oxides [34]. Furthermore, the correlation coefficient (R^2^) for the intra-particle diffusion model is lower than those obtained from the pseudo-second-order model, indicating that the adsorption kinetics are better described by the latter.

#### 3.2.4. Adsorption Isotherms

To better explain the interaction between the adsorbate and the filter paper-templated Mg-Al metal oxides, the Freundlich and Langmuir adsorption isotherm models were used to fit the isotherm parameters for the adsorption of sulfonated lignite. In this work, the Langmuir (Equation (6)) and Freundlich (Equation (7)) models were used to analyze the equilibrium adsorption data. The linear expressions of these models are as follows [35,36]:

(6) $$\frac{C_{e}}{q_{e}}=\frac{1}{q_{\max}K_{L}}+\frac{C_{e}}{q_{\max}}$$

(7) $$\ln q_{e}=\ln K_{F}+\frac{1}{n}\ln C_{e}$$

where *K_L_* is the Langmuir constant, *K_F_* is the Freundlich constant related to the energy of adsorption and adsorption capacity, and *q_max_* (mg·g^−1^) is the theoretical maximum adsorption capacity.

**Figure 11:**
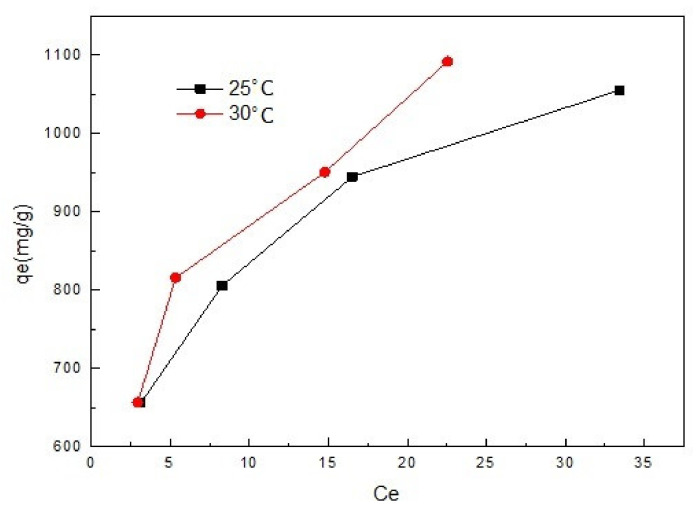
Equilibrium adsorption at different temperatures.

The influence of temperature on controlling the strength of the adsorptive forces between the adsorbent and adsorbate molecules plays an important role. The equilibrium data of sulfonated lignite adsorption by Mg-Al metal oxides at temperatures of 298 and 303 K are shown in Figure 11. It can be seen that as the reaction temperature rises to 30 °C, the maximum adsorption capacity of the filter paper-templated Mg-Al metal oxides for sulfonated lignite is 1189.29 mg/g. The results show that the adsorption capacity of the sulfonated lignite is favored by higher temperatures, and thus the adsorption process is endothermic, possibly partly due to the sulfonated lignite molecules moving around more and penetrating more deeply into the surface and pores of the Mg-Al metal oxides at increasing temperatures.

**Figure 12:**
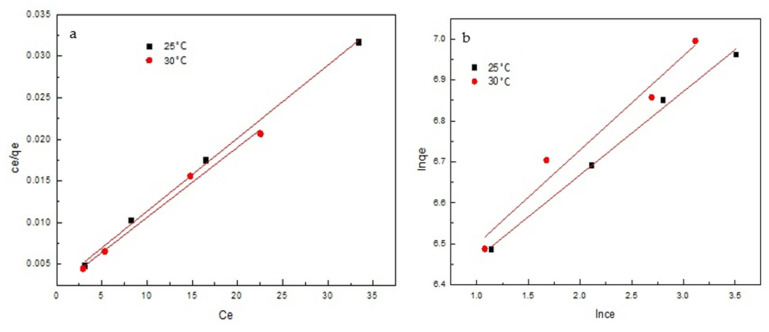
The Langmuir (**a**) and Freundlich (**b**) models for the adsorption of sulfonated lignite by filter paper-templated Mg-Al metal oxides at 25 and 30 °C.

The isotherm constants of the Langmuir and Freundlich isotherms, presented in Table 4, were calculated from the slope and intercept of the linear plot obtained in Figure 12a,b respectively. The correlation coefficient values obtained for the Langmuir isotherm were all above 0.99, indicating that the Langmuir model was more qualified to explicate the adsorption isotherm of sulfonated lignite by filter paper-templated Mg-Al metal oxides, and hence that the adsorption of sulfonated lignite by metal oxides may be monolayer adsorption [37]. The maximum adsorption capacity determined from the Langmuir model was 1189.29 mg/g for a temperature of 303 K. It is known that the constant *R*_L_ in the Langmuir equation is an important index for measuring the stability of the adsorbent/adsorbate complex. *R*_L_ = 1 represents linear behavior; 0 < *R*_L_ < 1, favorable to adsorption; *R*_L_ = 0, irreversible [38]. In this study, the separation factor *R*_L_ of the adsorption was found to be over a range of rather low values (max. 0.0149), and thus, the Langmuir isothermal model can better describe the adsorption process of sulfonated lignite by filter paper-templated Mg-Al metal oxides.

#### 3.2.5. Adsorption Thermodynamics

**Figure 13:**
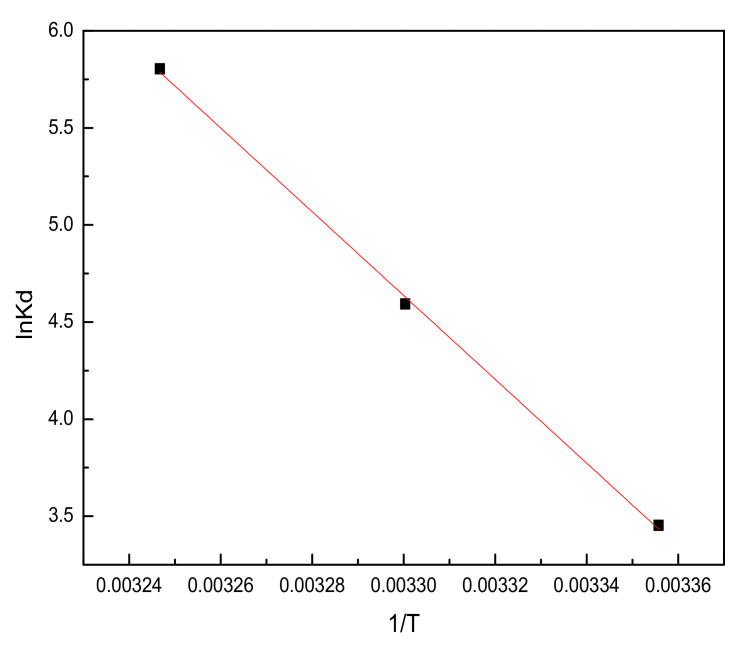
Effect of temperature on the adsorption of sulfonated lignite by filter paper-templated Mg-Al metal oxides (initial sulfonated lignite concentration: 350 mg/L; adsorbent dosage: 0.015; initial pH: 7; stirring time: 12 h).

Thermodynamic studies can be used to determine the energy changes in the adsorption process, and are highly significant for practical applications of an adsorption process. The thermodynamic parameters Gibbs free energy (ΔG, kJ·mol^−1^), enthalpy (ΔH, kJ·mol^−1^), and entropy (ΔS, kJ·mol^−1^·K^−1^) were calculated using the following equations [39]:

(8) $$\Delta G=-RT\ln K_{d}$$

(9) $$\ln K_{d}=\frac{\Delta S}{R}-\frac{\Delta H}{RT}$$

(10) $$K_{d}=\frac{q_{e}}{C_{e}}$$

where ΔG represents the standard Gibbs free energy change, *R* (8.314 J/mol k) is the universal gas constant, *T* is the absolute temperature (K), and *K_d_* is the distribution coefficient. ΔH and ΔS can be calculated from the slope and intercept of the Van ’t Hoff plots of ln*K_d_* versus 1/T (Figure 13).

The results obtained are summarized in Table 5. It can be seen that the ∆G values are negative, and increased in their absolute values with temperature, becoming increasingly negative with the increase in temperature (−8.51, −11.66, and −14.81 kJ/mol at 298, 303, and 308 K, respectively), indicating that high temperature facilitated the adsorption of sulfonated lignite by filter paper-templated metal oxides. Moreover, the positive value of ∆H indicates that the adsorption of sulfonated lignite is an endothermic process, which is consistent with the phenomenon of increased adsorption capacities with increasing temperature, as commented upon earlier in connection with Figure 11. The positive values of entropy indicate increasing freedom of movement of lignite at the solid/solution interface upon adsorption [40].

#### 3.2.6. Desorption and Regeneration

**Figure 14:**
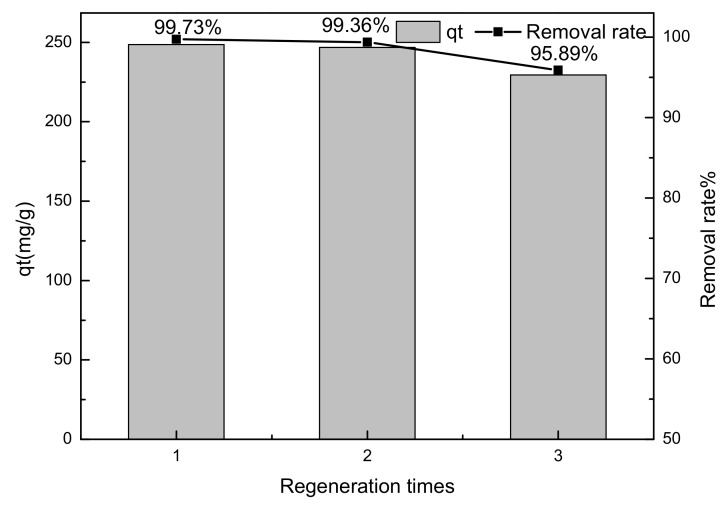
Influence of regeneration cycle on the adsorption of sulfonated lignite by filter paper-templated Mg-Al metal oxides (initial sulfonated lignite concentration: 200 mg/L; adsorbent dosage: 0.015; initial pH: 7; temperature set to 25 °C; stirring time: 12 h).

The stabilization and reutilization of adsorbents are some of the most important factors in the practical application of adsorbing materials, and they play an important role in the treatment of oilfield wastewater. Experiments were performed to investigate the activity on reuse, in which the adsorbent was regenerated by adding it to 1 mol/L, 50 mL of sodium carbonate solution under stirring at room temperature for 12 h. Then, the adsorbent was washed to neutral by deionized water, dried in a drying oven at 80 °C, calcined at 500 °C for 4h, and collected for reuse. It can be seen from Figure 14 that after three such cycles, the adsorption efficiency of Mg-Al metal oxides remained at 95.89%, indicating its good regeneration ability.

### 3.3. Formation Mechanism

The FTIR spectrum of sulfonated lignite adsorbed by Mg-Al metal oxides was investigated in order to propose a possible mechanism for the adsorption process. On comparing the FTIR spectra of Mg-Al metal oxides before and after sulfonated lignite adsorption, it is seen that the absorption band of Mg-Al metal oxides at 3445 cm^−1^, corresponding to the O-H stretching vibrations of hydrogen-bonded hydroxyl, is slightly shifted to 3448 cm^−1^, indicating that hydroxyl groups of Mg-Al metal oxides play an important role in the adsorption process, which we ascribe to the formation of hydrogen bonds by their interaction with the hydroxyl of sulfonated lignite. The peak shift from 1380 cm^−1^ to 1361 cm^−1^ could be ascribed to the intercalation of –SO_3_^−^ of sulfonated lignite. Moreover, after adsorption, the characteristic band of –SO_3_^−^ near 1035 cm^−1^ was very clear, which was clear evidence of the adsorption of sulfonated lignite by Mg-Al metal oxides.

Furthermore, calcined MgAl-LDHs have the ability to reconstruct their original layered structure after adsorption (“memory effect”); the XRD pattern of Mg-Al metal oxides/sulfonated lignite (Figure 4b) also exhibited the characteristic diffractions of hydrotalcite, indicating that the MgAl-LDHs were successfully reconstructed after the adsorption of sulfonated lignite. Moreover, the interlayer spacing of Mg-Al metal oxide after the adsorption of sulfonated lignite (0.75 nm) is larger than the interlayer spacing of MgAl-LDH (0.75 nm), indicating that layer expansion takes place upon adsorption. Therefore, in the reconstitution process, –SO_3_^−^ is intercalated into the layered structure, and CO_3_^2−^ ions are released simultaneously.

**Figure 15:**
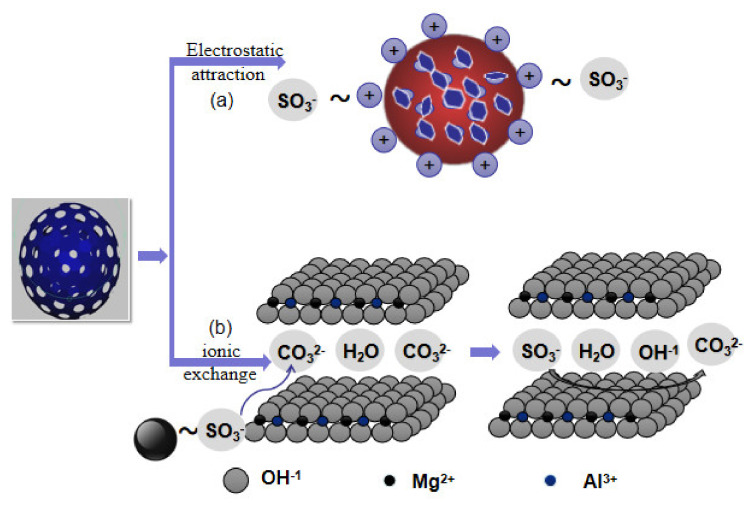
A possible mechanism for the adsorption of sulfonated lignite by biomorphic filter paper-templated Mg-Al metal oxides; (**a**) electrostatic attraction; (**b**) ionic exchange.

In conclusion, it can be speculated that the adsorption mechanism of Mg-Al metal oxides for sulfonated lignite is mainly involved in two processes of electrostatic attraction (Figure 15a) and ion exchange (Figure 15b).

## 4. Conclusions

In summary, a straightforward method for successfully synthesizing a series of Mg-Al metal oxides by using filter paper as a biotemplate has been developed. XRD, FTIR, SEM, and N_2_ adsorption–desorption analyses were applied to characterize and investigate the physicochemical properties of the synthesized samples. The obtained metal oxides all replicated the original macroarchitecture of the filter paper and had a hierarchically porous structure whose specific surface area was 178.84 mg/g. In addition, upon dosing 50 mL of 200 mg/L sulfonated lignite solution with 0.3 g/L of the adsorbent at a temperature of 298 K, the templated Mg-Al metal oxides showed improved adsorption performance, with 99.37% adsorption efficiency for sulfonated lignite. The reaction results were in accordance with the pseudo-second-order kinetic model and the Langmuir isotherm model. The thermodynamic parameters of ∆G and ∆H indicate that the adsorption of sulfonated lignite by Mg-Al metal oxides is a spontaneous endothermic process, and ∆S > 0, i.e., there is an increase in entropy during adsorption. This study provides a feasible and simple approach for the preparation of a high-performance material for oilfield wastewater treatment in a cost-effective way.

## Figures and Tables

**Table 1:** BET specific surface area (SSA), pore volume, and pore diameter of different samples.

Sample	BET Surface Area (m^2^/g)	Pore Volume (cm^3^/g)	Pore Diameter (nm)
Filter paper-templated Mg-Al metal oxides	178.84	0.3177	7.10
Without biotemplate	117.04	0.1879	6.42

**Table 2:** Adsorption kinetic parameters of the pseudo-first-order and pseudo-second-order models for the adsorption of sulfonated lignite by filter paper-templated Mg-Al metal oxides.

Concentration		Pseudo-First-Order Model			Pseudo-Second-Order Model		
*C*_0_	*qe,exp*	*qe,cal*	*K*_1_ *min^−^* ^1^	*R* ^2^	*qe,cal*	*K*_2_ *min^−^* ^1^	*R* ^2^
100	249.88	131.63	0.0089	0.817	268.82	0.912 × 10^−4^	0.997
150	373.43	193.33	0.01054	0.8325	400.00	0.678 × 10^−4^	0.994
200	498.43	264.36	0.01231	0.925	520.83	0.886 × 10^−4^	0.999

**Table 3:** Adsorption kinetic parameters of the intra-particle diffusion model for the adsorption of sulfonated lignite by filter paper-templated Mg-Al metal oxides.

Concentration	Intra-Particle Diffusion Model					
*C*_0_	*K*_d1_	*K*_d2_	*C*_1_	*C*_2_	*R*_1_^2^	*R*_2_^2^
100	5.742	1.378	127.84	217.33	0.516	0.436
150	3.204	0.490	251.42	361.91	0.809	0.698
200	10.110	1.257	312.66	469.61	0.995	0.698

**Table 4:** Adsorption isotherm parameters of Langmuir and Freundlich models for the adsorption of sulfonated lignite by filter paper-templated Mg-Al metal oxides.

Temperature	Langmuir Isotherm Model				Freundlich Isotherm Model		
*T* (°C)	*q_max_* (mg/g)	*K_L_* (L/mg)	*R_L_*	*R* ^2^	*K_F_* (mg/g)(L/mg)^1/n^	1/*n*	*R* ^2^
25	1139.56	0.34	0.0083–0.0149	0.997	523	0.2039	0.992
30	1189.29	0.38	0.0075–0.0129	0.99	527.63	0.2301	0.953

**Table 5:** Thermodynamic parameters for the adsorption of sulfonated lignite onto filter paper-templated Mg-Al metal oxides.

Temperature (K)	Δ*G* (KJ·mol^−1^)	Δ*H* (KJ·mol^−1^)	Δ*S* (J·mol^−1^·K^−1^)
298	−8.51	179.43	630.67
303	−11.66		
308	−14.81		

## Data Availability

The data presented in this study are available on request from the corresponding authors.
